# Surface Roughness Measurement on a Wing Aircraft by Speckle Correlation

**DOI:** 10.3390/s130911772

**Published:** 2013-09-05

**Authors:** Félix Salazar, Alberto Barrientos

**Affiliations:** Departamento de Física Aplicada (FARN), ETSI Minas, Universidad Politécnica de Madrid, C/Ríos Rosas 21, 28003 Madrid, Spain; E-Mail: alberto_barrientos@hotmail.com

**Keywords:** roughness measurement, speckle, non-contacting method, micro-detection

## Abstract

The study of the damage of aeronautical materials is important because it may change the microscopic surface structure profiles. The modification of geometrical surface properties can cause small instabilities and then a displacement of the boundary layer. One of the irregularities we can often find is surface roughness. Due to an increase of roughness and other effects, there may be extra momentum losses in the boundary layer and a modification in the parasite drag. In this paper we present a speckle method for measuring the surface roughness on an actual unmanned aircraft wing. The results show an inhomogeneous roughness distribution on the wing, as expected according to the anisotropic influence of the winds over the entire wing geometry. A calculation of the uncertainty of the technique is given.

## 1. Introduction

Numerous examples are known in which the surface roughness plays an important role in the operation and performance of all types of technological systems. In aeronautical engineering the use of specialty paints (coating), whose aim is to minimize the friction of the object relative to the fluid in which it moves, and the ice build-up and pollution on the wings of an aircraft are some examples in which the control of the roughness is important. In all these and many other cases, the increase in roughness can lead to significant negative effects. For these reasons, among others, the roughness and its effects have been investigated in-depth for a long time.

One specific case which we are interested is to study of the erosion (damage) on the wings of an actual aircraft, due to the mechanical influence of the wind and other factors that contribute to the increase of roughness. Taking into account the geometrical characteristics of a wing (length and cross-section), flight conditions and erosive effect of the wind, damage will be distinct on different parts of the airfoil. As a consequence, a non-homogeneous roughness distribution must appear.

An airfoil-shaped body which moves through a fluid produces an aerodynamic force [1]. The component of this force that is perpendicular to the direction of motion is called lift, and the component parallel to the direction of motion, which opposes body velocity, is called drag. Thus, the latter force (drag) is directly influenced by the body surface roughness and lift is affected in turn. In general, for flying it is desirable to reduce all drag (parasite and induced) to a minimum.

In fluid mechanics the layer of fluid in the immediate vicinity of a bounding surface is known as the boundary layer. In this area the factor that creates the majority of drag experienced by the boundary body is the fluid viscosity. One problem when increasing wing surface roughness is the change in the boundary layer flow of the airfoil, leading to a premature transition of this layer, which results in early turbulent separation. Any change in its contour will cause a detachment of the boundary layer at lower angles of attack. As a result a greater overall drag value, that is to say, the force opposing the advance of the plane, will be obtained. Therefore, because an aircraft having rough wings must fly with higher angles of attack to maintain lift (the boundary layer separation occurs before), drag force will be greater than in the case of the smooth airfoil. Consequently, fuel consumption will increase, as the airplane it will have to counter increased drag. Notwithstanding, this negative influence is not only limited to the wings of an aircraft.

Direct and indirect effects on the different mechanical elements have been extensively studied to date, so a similar effect may be found when studying wind turbines and other machines. Thus, the roughness modification of the rotor blade airfoils because of contamination also leads to power loss and to an increase of the boundary layer thickness [2–4]. In general, frictional drag due to surface roughness is an important task in fluid engineering research [5].

In this article the measurement of the different degrees of surface roughness on a wing of an unmanned aircraft (MILANO unmanned aircraft-UAV) is presented. Traditionally the most used method has been the profilometer technique. However, it has been revealed that this procedure is not useful to apply to the case of an entire airfoil wing considering that this system is based on the registration of the surface topography of a small sample by means of a type stylus. In order to avoid this drawback, an optical method, which is based on the speckle phenomenon, has been employed. There are distinct speckle procedures for measuring roughness [6–11]. For the present investigation the angular speckle correlation (ASC) is chosen [7,11]. This technique, even though requires an accurate alignment of the optical light beams, and it is very sensitive to environmental vibrations, allows us easily and accurately to measure the non-contact roughness on all parts of the wing.

## 2. Theoretical Concepts and Basic Equations

In the field of the speckle techniques, the two most important set-ups used are the following: (1) the angular speckle correlation method (ASC) and (2) the spectral speckle correlation technique (SSC) [7,11]. The first one is obtained by using an optical system in which a laser beam under an angle *α* is directed onto the rough surface that is to be examined. In order to carry out the speckle correlation, a second exposure is obtained in the same manner, but the incidence angle of the light beam is changed by amount *δα*. The second option is similar but instead to vary the incidence angle, the beam remains constant while only changing the wavelength of the laser. This article deals with the ASC method, thus we will refer only to this technique.

Assuming a plane surface (macroscopically) and a Gaussian distribution for its surface heights, and neglecting multiple scattering and shadowing effects, it can be demonstrated that, for a focal length given the correlation of two speckle patterns in the Fourier plane corresponding to two incidence angles of the laser beam is [7,11]:

(1) $$C\left(\alpha_{i},\alpha_{o},\delta \alpha ,\Delta \xi ,\Delta \eta \right)=\exp\left[-{\left(\left(\sin\alpha_{i}\delta \alpha +\sin\alpha_{o}\frac{\Delta \xi }{f}\right)k\sigma_{h}\right)}^{2}\right]\exp\left[-{\left(\frac{Lk}{2f}\right)}^{2}\left({\left(\Delta \xi -\frac{\cos\alpha_{i}}{\cos\alpha_{0}}f\delta \alpha \right)}^{2}+{\left(\Delta \eta \right)}^{2}\right)\right]$$

where *k* the wave number of the monochromatic beam, *f* the focal length of the lens, *σ_h_* is the variance of the surface, *L* is the half-width of the laser, and *α_i_* and *α_o_* the angles of incidence and observation, respectively. *ξ* and *η* refer to the coordinate system on the detector (Fourier plane). By introducing all parameters corresponding to a specific set-up in Equation (1), the measurement of the roughness may be conducted. However, in order to carry out the measurement as easily as possible and, on the other hand, minimizing the uncertainty, we can simplify this equation if the following conditions apply:

(2) $$\Delta \xi -\frac{\cos\alpha_{i}}{\cos\alpha_{0}}f\delta \alpha =0,$$

and,

(3) Δη=0.

Substituting both results in Equation (1) leads to:

(4) $$C\left(\alpha_{i},\alpha_{o},\delta \alpha ,\Delta \xi ,\Delta \eta \right)=\exp\left(-{\left(\left(\sin\alpha_{i}\delta \alpha +\tan\alpha_{o}\cos\alpha_{i}\delta \alpha \right)k\sigma_{h}\right)}^{2}\right)$$

In this paper we chose the condition *α_i_* = *α_o_* = *α*, then expression for the correlation becomes:

(5) $$C\left(\alpha ,\delta \alpha \right)=\exp(-{\left(2k\sin\alpha \delta \alpha \sigma_{h}\right)}^{2}),$$

which is easy to manipulate because it depends directly on *σ_h_*. Therefore, by measuring the correlation of two speckle pattern shifted by an amount Δ*ξ*=*fδα*, the roughness of a surface may be determined as follows:

(6) $$\sigma_{h}=\frac{\lambda \sqrt{-\ln C\left(\alpha ,\delta \alpha \right)}}{4\pi \delta \alpha \sin\alpha }.$$

## 3. Uncertainty by the ASC Method

In this section the systematic uncertainty of the measurement by the ASC technique is computed. With this aim the basic theory of uncertainties [12] is applied with the formula *σ_h_*, which for this particular case adopts the form:

(7) $$I_{s}(\sigma_{h})=\sum_{j=1}^{N}\left|\frac{\partial \sigma_{h}}{\partial x_{j}}\right|I_{s}(x_{j}),$$

where *x_j_* are *λ*, *α* and *δα*. Taking into account that $\sigma_{h}=\frac{\lambda \sqrt{-\ln C}}{4\pi \delta \alpha \sin\alpha }$, applying Equation (7) to this equation, we obtain:

(8) $$I_{s}\left(\sigma_{h}\left(\lambda ,\alpha ,\delta \alpha \right)\right)=\left|\frac{\sqrt{-\ln C}}{4\pi \delta \alpha \sin\alpha }\right|I_{s}(\lambda )+\left|\frac{\lambda \sqrt{-\ln C}}{4\pi {\left(\delta \alpha \right)}^{2}\sin\alpha }\right|I_{s}(\delta \alpha )+\left|\frac{\lambda \cos\alpha \sqrt{-\ln C}}{4\pi \delta \alpha {\left(\sin\alpha \right)}^{2}}\right|I_{s}(\alpha ),$$

and the relative uncertainty:

(9) $$\frac{I_{s}(\sigma_{h})}{\sigma_{h}}=\left|\frac{I_{s}(\lambda )}{\lambda }\right|+\left|\frac{I_{s}(\delta \alpha )}{\delta \alpha }\right|+\left|\frac{I_{s}(\alpha )}{tg\alpha }\right|.$$

For the case analyzed *I_s_*(*δα*) = 1.2 × 10^−3^ rad. On the other hand, assuming *I_s_*(*λ*) = 0.1 nm, *α*=45° and *I_s_*(*α*) = 1.7 × 10^−2^ rad, Equation (7) yields:

(10) $$\frac{I_{s}(\sigma_{h})}{\sigma_{h}}=1.6\times 10^{-4}+7.4\times 10^{-2}+1.7\times 10^{-2}\approx 9.2\times 10^{-2}=9.2\%.$$

It may be observed that the second term contributes more to the uncertainty than the rest. It corresponds to the angular difference between incident beams. To reduce this uncertainty and improve the measurement, a higher precision when measuring the angle is necessary. However, with this set-up and experimental values the precision of the technique is 0.1 μm.

The above calculation is only valid if the angles of incidence and observation are the same. However, if one of them is different, then the Equation (8) must be modified. In this case, following the same way as before we can write:

(11) $$U_{s}(\sigma_{h})=\sum_{j=1}^{N}\left|\frac{\partial \sigma_{h}(\lambda ,\delta \alpha ,\alpha_{0},\alpha_{i})}{\partial x_{j}}\right|U_{s}(x_{j}),$$

where more variables appear. By calculating partial derivatives it leads to:

(12) $$\frac{U_{s}(\sigma_{h})}{\sigma_{h}}=\left|\frac{U_{s}(\lambda )}{\lambda }\right|+\left|\frac{U_{s}(\delta \alpha )}{\delta \alpha }\right|+\left|\frac{U_{s}(\alpha_{i})}{\left(\frac{\sin\alpha_{i}+tg\alpha_{0}\cos\alpha_{i}}{\cos\alpha_{i}-tg\alpha_{0}\sin\alpha_{i}}\right)}\right|+\left|\frac{U_{s}(\alpha_{0})}{\frac{\cos^{2}\alpha_{0}\left(\sin\alpha_{i}+tg\alpha_{0}\cos\alpha_{i}\right)}{\cos\alpha_{i}}}\right|.$$

This expression may be simplified to give:

$$\frac{U_{s}(\sigma_{h})}{\sigma_{h}}=\left|\frac{U_{s}(\lambda )}{\lambda }\right|+\left|\frac{U_{s}(\delta \alpha )}{\delta \alpha }\right|+\left|\frac{U_{s}(\alpha_{i})}{\left(\frac{\sin\alpha_{i}+tg\alpha_{0}\cos\alpha_{i}}{\cos\alpha_{i}-tg\alpha_{0}\sin\alpha_{i}}\right)}\right|+\left|\frac{U_{s}(\alpha_{0})}{\cos^{2}\alpha_{0}\left(\tan\alpha_{i}+tg\alpha_{0}\right)}\right|.$$

Let us suppose that the observation angle (45°) is maintained, but the incidence angle changes. For this specific case the equality becomes:

(13) $$\begin{array}{l} \frac{U_{s}(\sigma_{h})}{\sigma_{h}}=\left|\frac{U_{s}(\lambda )}{\lambda }\right|+\left|\frac{U_{s}(\delta \alpha )}{\delta \alpha }\right|+\left|\frac{U_{s}(\alpha_{i})}{\left(\frac{\sin\alpha_{i}+tg45\cos\alpha_{i}}{\cos\alpha_{i}-tg45\sin\alpha_{i}}\right)}\right|+\left|\frac{U_{s}(\alpha_{0})}{\cos^{2}45\left(\tan\alpha_{i}+tg45\right)}\right|=\\ \left|\frac{U_{s}(\lambda )}{\lambda }\right|+\left|\frac{U_{s}(\delta \alpha )}{\delta \alpha }\right|+\left|\frac{U_{s}(\alpha_{i})}{\left(\frac{\sin\alpha_{i}+\cos\alpha_{i}}{\cos\alpha_{i}-\sin\alpha_{i}}\right)}\right|+\left|\frac{U_{s}(\alpha_{0})}{\left(\frac{1+\tan\alpha_{i}}{2}\right)}\right|. \end{array}$$

By comparing this result with Equation (9) we observe that the two first terms are the same. Thus, in order to investigate whether this uncertainty *U_s_*(*σ_h_*) is greater than *I_s_*(*σ_h_*), we can focus our attention to the last summands of Equation (13). In fact, as *U_s_*(*α_i_*)=*U_s_*(*α_o_*)=*I_s_*(*α*), for the first uncertainty Equation (9) to be smaller than the second one, the following inequality must apply:

(14) $$\left|\frac{1}{tg45}\right|<\left|\frac{1}{\left(\frac{\sin\alpha_{i}+\cos\alpha_{i}}{\cos\alpha_{i}-\sin\alpha_{i}}\right)}\right|+\left|\frac{1}{\left(\frac{1+\tan\alpha_{i}}{2}\right)}\right|.$$

However, even though this inequality is difficult to resolve, as the first term of the second member always adds, this relation is always fulfilled if:

(15) $$\left|\frac{1}{\left(\frac{1+\tan\alpha_{i}}{2}\right)}\right|>\left|\frac{1}{\tan45}\right|\Rightarrow \frac{1+\tan\alpha_{i}}{2}<1,$$

that is to say, *α_i_*<(*π*/4). This result shows that the best disposition for the layout in order to reduce the uncertainty of the method is to use an angle of 45°.

## 4. Experimental Results

**Figure 1.**
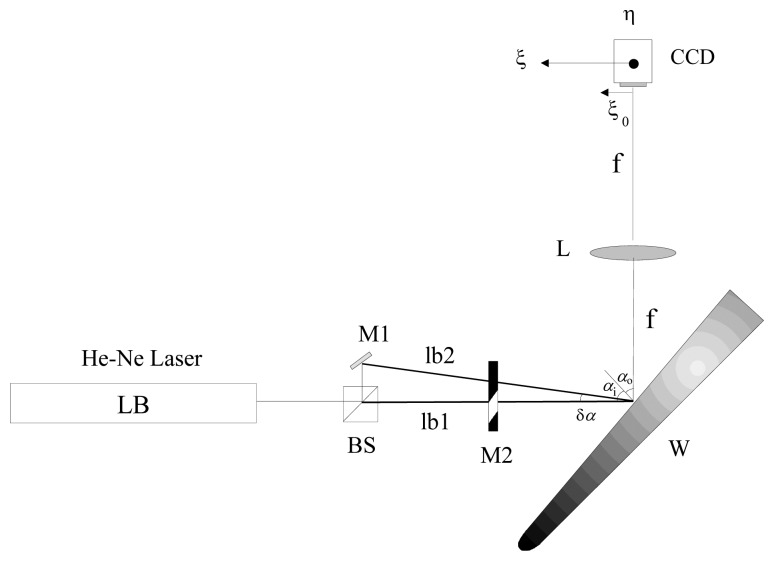
Layout. (**a**) LB, laser beam; (**b**) BS, beam splitter; (**c**) M1, Mirror; (**d**) M2, gradual mirror; (**e**) W, wing (sample); (**f**) L, lens; (**g**) CCD, detector; (**h**) *δα*, *d*ifference angle of the two beams; (**i**) *f*, focal length; (**j**) *ξ* and *η*, CCD coordinate system; (**k**) *ξ*_0_, lateral displacement of the CCD camera.

**Figure 2.**
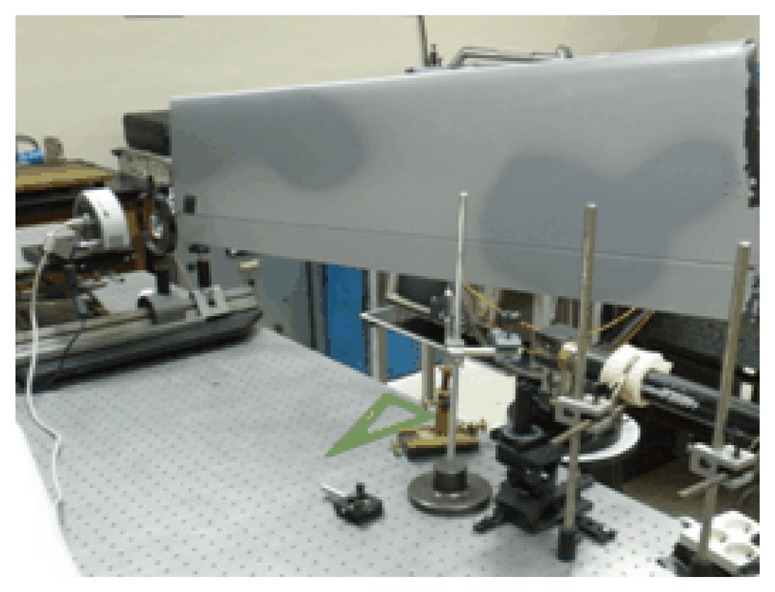
Experimental set-up employed. The picture corresponds to the measurement at a point located on the extreme of the wing flap (the length of the wing is 1.30 m, approximately).

**Figure 3.**
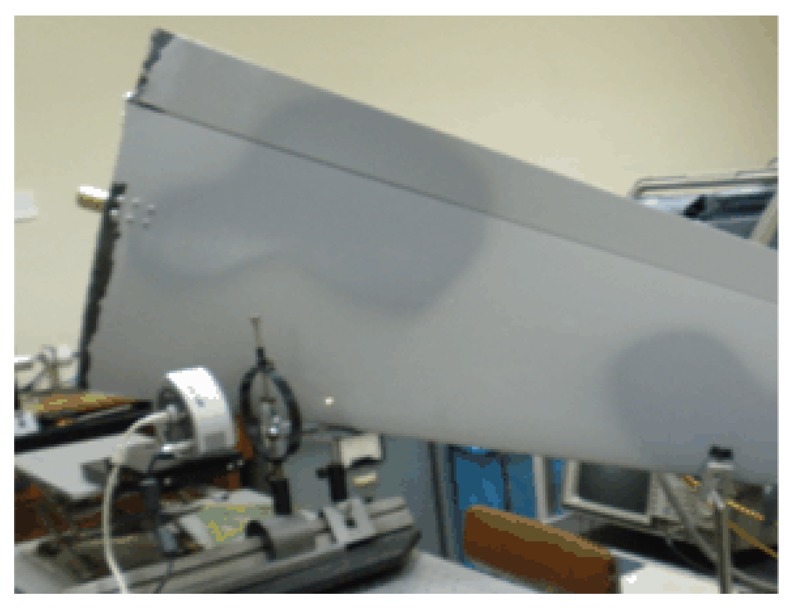
Experimental device. In this case the photograph shows a point located on the middle of the wing near the leading edge.

In order to investigate the effect of the wind on the surface of an actual aircraft wing, measurement of the roughness on different parts of the wing were carried out. To perform the experiments a device as shown in Figure 1 was employed (see also Figures 2 and 3). A He-Ne laser LB was directed to a beam splitter (BS) for separating the initial beam in two parts. The first one (lb1) passes through a variable reflective mirror (M2) and strikes on the wing (sample). The second (lb2) is first reflected by the mirror M1 and also transmitted through the variable mirror M2 and, after that, impacting on the sample. The use of a graduated mirror allows us to adjust the intensities of both speckle patterns on the detector avoiding errors when calculating the correlation. The light scattered by the rough surface in the two exposures is separately collected by the lens and focused on the CCD array (Figure 5).

**Figure 4.**
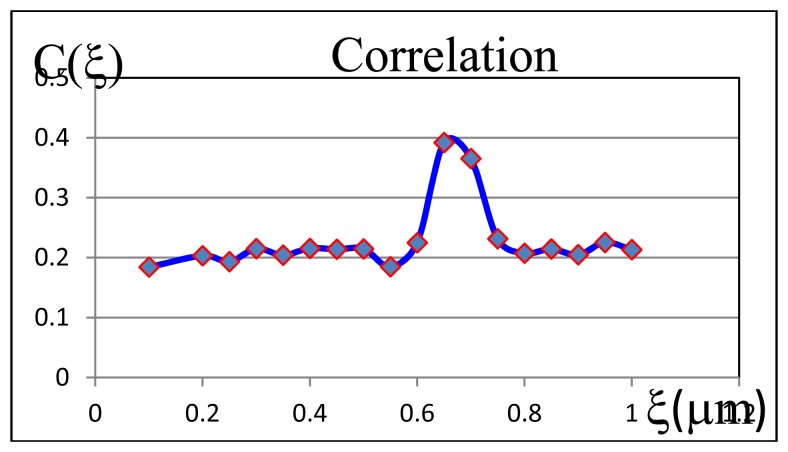
Experimental plot of the correlation C versus displacement (in μm) of the CCD camera. Notice that the maximum is reached away of the first exposure where the camera was not yet displaced.

**Figure 5.**
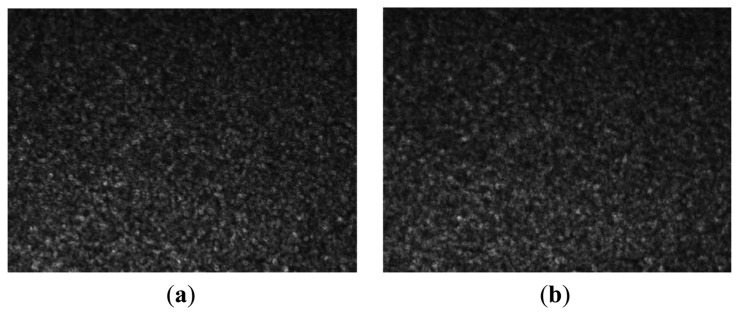
Speckle patterns corresponding to the maximum correlation. (**a**) First exposure for the laser beam (lb1) for an incidence of 45°. (**b**) Speckle field picture of the second beam (lb2) when the CCD camera is displaced by an amount Δ*ξ*=*fδα*. The pictures correspond to a camera of 658 × 496 pixels (8.5 × 6.5 mm^2^).

Owing to the different angle of incidence, the two speckle patterns are displaced [see Equation (2)]. It means that for calculating the maximum correlation of the two fields we must displace the detector by a quantity of Δ*ξ*=*fδα* [7,11]. In principle this procedure may be carried out directly with these two patterns only. However, to be sure these Equations (2) and (3) are met, it is advisable to register a series of speckle patterns for the second exposure, each of which is taken by moving the CCD camera in small steps. Thus, through this procedure we have confidence that the maximum correlation is found. Figure 4 shows the correlation corresponding to the series of two speckle pictures obtained for one of the examined points of the airfoil. Observe that the maximum is found far away of the first exposure.

**Figure 6.**
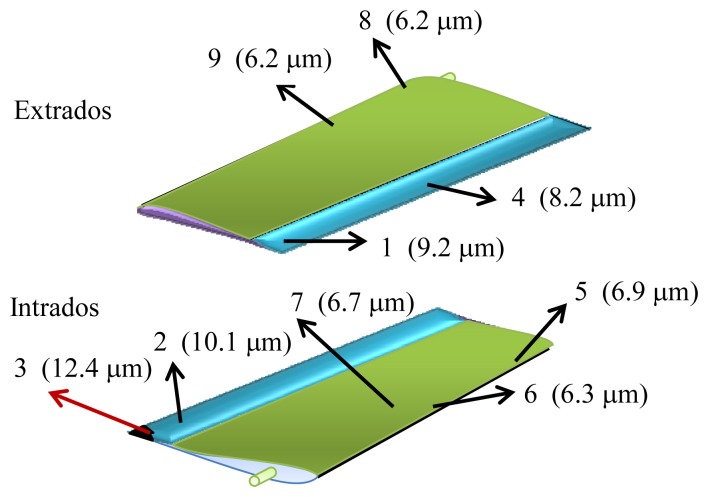
This picture shows the roughness at each point measured on the airfoil. Point 3 is a special case because it does not have coating.

All experiments were made for *λ*=632.8 nm, *f* = 8.5 cm, *α_i_*= *α_o_*= 45°, and *δα* = 1.9°. The experimental results obtained for the roughness *σ_h_* are represented in Table 1. In order to resume this table, a picture of the wing with all points examined and their roughness is represented in Figure 6.

The initial roughness of the wing was 2 μm, and then these results show that after many hours of flight all parts deteriorate, but differently, and on places near the leading edge, both above and below the aircraft wing, the wear is less than on the flap. The interpretation of all these numerical values can be understood by means of fluid mechanics.

In effect, near the leading edge of the wing up to the shoulder, a favorable pressure gradient exists and the airflow is laminar [1]. This way of interacting for the air molecules with this part of the airfoil is almost isotropic, thus the mechanical action against the wing surface must be alike (points 5, 6, 7, and 8). As a result the induced roughness due to wear remains similar for upper and lower parts of the airfoil (about 6 μm). On the contrary, when the flow displaces from the shoulder to the rear surface of the wing, an unfavorable pressure gradient appears, which leads to a change in the airflow and in the boundary layer, which becomes turbulent. As it is well known, when turbulence is present the particles of the fluid describe a chaotic motion in very complicated trajectories. These particles within the wake fluctuate quickly, bombarding the flap surface of the airfoil. This fact explains the reason why the roughness on this area of the wing is greater than on other parts studied. On the other hand, when examining the results along the flap we observe that the roughness at point 2 (intrados) is greater than at points 1 and 4 (extrados). Again, from the point of view of fluid mechanics it may be justified. So, it is well known that the parts of an aircraft (and not only in this geometry) where surfaces join, increases turbulence. If we examine point 2 we notice that located near the intersection of the fuselage and wing, where both surfaces join, an angle forms between them. As a consequence a turbulent wind regime must appear and, by the same reasoning aforementioned, the erosion on this region must be greater than for other flap areas. In the same way, comparing the points 1 and 4 a grater roughness at 1 is observed, indicating that the occurrence of airfoil wingtip vortices has a greater influence in this part than in the middle of the flap. A special result refers to point 3 where the greatest roughness is detected. In this case, because of the eroding wind action, the area studied did not have coating, and such the roughness must be greater (12.4 μm). This value confirms the above explanation for emerging turbulences on regions where surfaces join. To conclude this section it is interesting to observe that, due to the precision of the ASC technique (see Section 2) and the values of the roughness obtained, the methodology seems to be quite adequate for measuring the problem studied.

## 5. Conclusions

In this paper a study of the roughness on an actual unmanned aircraft wing is presented. By using the angular speckle correlation technique (ASC), the degree of damage at different small areas of the airfoil is measured. The study reveals a non-homogeneous resultant mechanical effect of the wind, and other factors, on the surface microstructure. As expected, because of the apparition of turbulences over the flap and near to the intersection between the wing and the fuselage, the erosion is greater over these areas than on the leading edge, where the airflow is almost laminar. Besides, a study of the uncertainty of the technique is made, and a recommendation for the best experimental set-up is given.

## Figures and Tables

**Table 1.** Experimental roughness obtained for the selected points of the aircraft wing.

**Loci**	**Position**	**Roughness *R*_q_(μm)**
1	Extrados	9.2
2	Intrados	10.1
3	Intrados	12.4
4	Extrados	8.2
5	Intrados	6.9
6	Intrados	6.3
7	Intrados	6.7
8	Extrados	6.2
9	Extrados	6.2
